# Supplementary material for: Estimation of the central 10-degree visual field using en-face images obtained by optical coherence tomography
Source: PLoS One. 2020 Mar 5;15(3):e0229867. doi: 10.1371/journal.pone.0229867 (PMC7058317; doi:10.1371/journal.pone.0229867)

PLOS ONE

Estimation of the central 10-degree visual field using en-face images obtained by optical coherence tomography

--Manuscript Draft--

**Manuscript Number:**	PONE-D-19-21988
**Article Type:**	Research Article
**Full Title:**	Estimation of the central 10-degree visual field using en-face images obtained by optical coherence tomography
**Short Title:**	Estimation of visual field with en-face OCT images
**Corresponding Author:**	Takeo Fukuchi<br>Graduated School of Medical and Dental Sciences, Niigata University<br>Niigata, Niigata JAPAN
**Keywords:**	swept-source OCT; en-face image; glaucoma; visual field
**Abstract:**	Purpose<br>To estimate the central 10-degree visual field of glaucoma patients using en-face images obtained by optical coherence tomography (OCT), and to examine its usefulness.<br><br>Patients and methods<br>Thirty-eight eyes of 38 patients with primary open angle glaucoma (POAG) were examined. En-face images were obtained by swept-source OCT (SS-OCT). Nerve fiber bundles (NFBs) on the en-face images at points corresponding to Humphrey Field Analyzer (HFA) 10-2 locations were identified with RGC displacement. Estimated visual fields were created based on the presence/absence of NFBs and compared to actual HFA10-2 data. κ coefficients were calculated between probability plots of visual fields and NFBs in en-face images.<br><br>Results<br>Actual HFA10-2 data and estimated visual fields based on en-face images were well matched: when the test points of <5%, <2%, and <1% of the probability plot in total deviation (TD) and pattern deviation (PD) of HFA were defined as points with visual field defects, the κ coefficients were 0.58, 0.64, and 0.66 in TD, respectively, and 0.68, 0.69, and 0.67 in PD. In eyes with spherical equivalent ≧ −6 diopters, κ coefficients for <5%, <2%, and <1% were 0.58, 0.62, and 0.63 in TD and 0.66, 0.67, and 0.65 in PD, whereas for the myopic group with spherical equivalent < −6 diopters, the values were 0.58, 0.69, and 0.71 in TD and 0.72, 0.71, and 0.71 in PD, respectively. There was no statistically significant difference in κ coefficients between myopic eyes, in which NFBs are not easily observed due to tigroid fundus, and non-myopic eyes.<br><br>Conclusions<br>NFB defects in en-face images were correlated with HFA10-2 data. Using en-face images obtained by OCT, the central 10-degree visual field was estimated, and a high degree of concordance with actual HFA10-2 data was obtained. This method may be useful for detecting functional abnormalities based on structural abnormalities.
**Order of Authors:**	ryu iikawa
	Tetsuya Togano
	Yuta Sakaue
	Aki Suetake
	Ryoko Igarashi
	Daiki Miyamoto
	Kiyoshi Yaoeda
	Masaaki Seki

Powered by Editorial Manager® and ProduXion Manager® from Aries Systems Corporation

	Takeo Fukuchi
**Additional Information:**	
**Question**	**Response**
**Financial Disclosure**<br><br>Enter a financial disclosure statement that describes the sources of funding for the work included in this submission. Review the submission guidelines for detailed requirements. View published research articles from *PLOS ONE* for specific examples.<br><br>This statement is required for submission and **will appear in the published article** if the submission is accepted. Please make sure it is accurate.<br><br>**Unfunded studies**<br>Enter: *The author(s) received no specific funding for this work.*<br><br>**Funded studies**<br>Enter a statement with the following details:<br>• Initials of the authors who received each award<br>• Grant numbers awarded to each author<br>• The full name of each funder<br>• URL of each funder website<br>• Did the sponsors or funders play any role in the study design, data collection and analysis, decision to publish, or preparation of the manuscript?<br>• **NO** - Include this sentence at the end of your statement: *The funders had no role in study design, data collection and analysis, decision to publish, or preparation of the manuscript.*<br>• **YES** - Specify the role(s) played.<br><br>* typeset	The author(s) received no specific funding for this work.
**Competing Interests**<br><br>Use the instructions below to enter a competing interest statement for this submission. On behalf of all authors, disclose any competing interests that could be perceived to bias this work—acknowledging all financial support and any other relevant financial or non-financial competing interests.	The authors have declared that no competing interests exist.

Powered by Editorial Manager® and ProduXion Manager® from Aries Systems Corporation

This statement **will appear in the published article** if the submission is accepted. Please make sure it is accurate. View published research articles from *PLOS ONE* for specific examples.<br><br>**NO authors have competing interests**<br><br>Enter: *The authors have declared that no competing interests exist.*<br><br>**Authors with competing interests**<br><br>Enter competing interest details beginning with this statement:<br><br>*I have read the journal's policy and the authors of this manuscript have the following competing interests: [insert competing interests here]*<br><br>* typeset	
**Ethics Statement**<br><br>Enter an ethics statement for this submission. This statement is required if the study involved:<br><br>• Human participants<br>• Human specimens or tissue<br>• Vertebrate animals or cephalopods<br>• Vertebrate embryos or tissues<br>• Field research<br><br>Write "N/A" if the submission does not require an ethics statement.<br><br>General guidance is provided below. Consult the submission guidelines for detailed instructions. **Make sure that all information entered here is included in the Methods section of the manuscript.**	This study was approved by the ethics committee of the Niigata University Graduate School of Medical and Dental Sciences and followed the tenets of the Declaration of Helsinki.

**Format for specific study types**<br><br>**Human Subject Research (involving human participants and/or tissue)**<br>• Give the name of the institutional review board or ethics committee that approved the study<br>• Include the approval number and/or a statement indicating approval of this research<br>• Indicate the form of consent obtained (written/oral) or the reason that consent was not obtained (e.g. the data were analyzed anonymously)<br><br>**Animal Research (involving vertebrate animals, embryos or tissues)**<br>• Provide the name of the Institutional Animal Care and Use Committee (IACUC) or other relevant ethics board that reviewed the study protocol, and indicate whether they approved this research or granted a formal waiver of ethical approval<br>• Include an approval number if one was obtained<br>• If the study involved *non-human primates*, add *additional details* about animal welfare and steps taken to ameliorate suffering<br>• If anesthesia, euthanasia, or any kind of animal sacrifice is part of the study, include briefly which substances and/or methods were applied<br><br>**Field Research**<br><br>Include the following details if this study involves the collection of plant, animal, or other materials from a natural setting:<br>• Field permit number<br>• Name of the institution or relevant body that granted permission	
**Data Availability**<br><br>Authors are required to make all data underlying the findings described fully available, without restriction, and from the time of publication. PLOS allows rare exceptions to address legal and ethical concerns. See the PLOS Data Policy and FAQ for detailed information.	Yes - all data are fully available without restriction

Powered by Editorial Manager® and ProduXion Manager® from Aries Systems Corporation

A Data Availability Statement describing where the data can be found is required at submission. Your answers to this question constitute the Data Availability Statement and **will be published in the article**, if accepted.<br><br>**Important:** Stating 'data available on request from the author' is not sufficient. If your data are only available upon request, select 'No' for the first question and explain your exceptional situation in the text box.<br><br>Do the authors confirm that all data underlying the findings described in their manuscript are fully available without restriction?	
**Describe where the data may be found in full sentences. If you are copying our sample text, replace any instances of XXX with the appropriate details.**<br><br>• If the data are **held or will be held in a public repository**, include URLs, accession numbers or DOIs. If this information will only be available after acceptance, indicate this by ticking the box below. For example: *All XXX files are available from the XXX database (accession number(s) XXX, XXX.).*<br>• If the data are all contained **within the manuscript and/or Supporting Information files**, enter the following: *All relevant data are within the manuscript and its Supporting Information files.*<br>• If neither of these applies but you are able to provide **details of access elsewhere**, with or without limitations, please do so. For example:<br><br>*Data cannot be shared publicly because of [XXX]. Data are available from the XXX Institutional Data Access / Ethics Committee (contact via XXX) for researchers who meet the criteria for access to confidential data.*<br><br>*The data underlying the results presented in the study are available from (include the name of the third party*	All relevant data are within the manuscript and its Supporting Information files.

and contact information or URL).<br>• This text is appropriate if the data are owned by a third party and authors do not have permission to share the data.<br><br>* typeset	
Additional data availability information:	

Manuscript Click here to download Manuscript manuscript.docx

Estimation of the central 10-degree visual field using en-face images obtained by optical coherence tomography

Ryu Iikawa, Tetsuya Togano, Yuta Sakaue, Aki Suetake, Ryoko Igarashi, Daiki Miyamoto, Kiyoshi Yaoeda, Masaaki Seki, Takeo Fukuchi*

Division of Ophthalmology and Visual Science, Graduate School of Medical and Dental Sciences, Niigata University, Niigata, Japan

*Corresponding author

E-mail: tfuku@med.niigata-u.ac.jp (TF)

Short title: Estimation of visual field with en-face OCT images

Abstract

Purpose

To estimate the central 10-degree visual field of glaucoma patients using en-face images obtained by optical coherence tomography (OCT), and to examine its usefulness.

Patients and methods

Thirty-eight eyes of 38 patients with primary open angle glaucoma (POAG) were examined. En-face images were obtained by swept-source OCT (SS-OCT). Nerve fiber bundles (NFBs) on the en-face images at points corresponding to Humphrey Field Analyzer (HFA) 10-2 locations were identified with RGC displacement. Estimated visual fields were created based on the presence/absence of NFBs and compared to actual HFA10-2 data. κ coefficients were calculated between probability plots of visual fields and NFBs in en-face images.

Results

Actual HFA10-2 data and estimated visual fields based on en-face images were well matched: when the test points of <5%, <2%, and <1% of the probability plot in total deviation (TD) and pattern deviation (PD) of HFA were defined as points with visual field defects, the κ coefficients were 0.58, 0.64, and 0.66 in TD, respectively, and 0.68, 0.69, and 0.67 in PD. In eyes with spherical equivalent $\geqq -6$ diopters, κ coefficients for <5%,

<2%, and <1% were 0.58, 0.62, and 0.63 in TD and 0.66, 0.67, and 0.65 in PD, whereas for the myopic group with spherical equivalent < −6 diopters, the values were 0.58, 0.69, and 0.71 in TD and 0.72, 0.71, and 0.71 in PD, respectively. There was no statistically significant difference in κ coefficients between myopic eyes, in which NFBs are not easily observed due to tigroid fundus, and non-myopic eyes.

Conclusions

NFB defects in en-face images were correlated with HFA10-2 data. Using en-face images obtained by OCT, the central 10-degree visual field was estimated, and a high degree of concordance with actual HFA10-2 data was obtained. This method may be useful for detecting functional abnormalities based on structural abnormalities.

Introduction

Glaucoma is a condition that involves distinctive changes in the optic nerve and visual field [1,2]. Because glaucoma is a disease characterized by functional and structural abnormalities of the eye, diagnosis and treatment of this condition requires accurate fundus imaging and determination of the visual field. In general, HFA24-2 is frequently used to evaluate the status of the visual function of patients with glaucoma, and examination of the visual field plays a very important role in evaluating vision-related quality of life (VR-QO[illegible] However, an area related to VR-QOL exists in the center of the visual field [3], and the correlation with the National Eye Institute 25-item Visual Function Questionnaire (NEI VFQ-25) score is higher in Humphrey Field Analyzer (HFA; Carl Zeiss Meditec, Dublin, CA, USA) 10-2 than HFA24-2 [4]; visual field defects within the central 10 degrees have a large influence on VR-QOL. In recent years, optical coherence tomography (OCT) has been used to detect structural fundus abnormality; in particular, the circumpapillary retinal nerve fiber layer (cpRNFL) is used in many cases for glaucoma diagnosis. On the other hand, in glaucoma patients with central visual field disorder in particular, misdiagnoses often arise in examinations using the cpRNFL or the HFA30-2/24-2 program. Therefore, HFA10-2 is necessary to evaluate VR-QOL [5-9]. However, it is difficult to perform HFA10-2 in all patients due to issues related to factors

such as fatigue and measurement time. In addition, because the visual field examination is a psychophysiological test, repeated examinations are necessary, the variation is large, and the result fluctuates in response to patients' psychological factors, physical condition, age, etc.

Hence, in this study we sought to estimate the central 10-degree visual field based on structural abnormality in OCT, which takes only a few seconds. This is the first report showing a correlation between NFB defects in en-face images and visual field defects in HFA10-2.

Patients and methods

Patients

This study was approved by the ethics committee of the Niigata University Graduate School of Medical and Dental Sciences and followed the tenets of the Declaration of Helsinki. Thirty-eight eyes of 38 patients with primary open angle glaucoma (POAG) were recruited consecutively, from Niigata University Medical and Dental Hospital between May 2012 and March 2015. If both eyes corresponded to the inclusion criteria in a patient, the eye with worse mean deviation (MD) was enrolled in the study. All glaucoma patients first underwent general ophthalmic examination, including refraction,

keratometry, assessment of visual acuity using a Landolt chart, assessment of intraocular pressure using a Goldman applanation tonometer, gonioscopy, slit-lamp examination, fundus examination, swept-source (SS) OCT (DRI OCT-1 Atlantis; Topcon, Tokyo, Japan), and visual field examination using the HFA30-2 or 24-2 and HFA10-2 SITA standard strategy. Diagnosis of POAG was made according to the guidelines of the Japan Glaucoma Society and the European Glaucoma Society.

Only patients with good reliability in HFA10-2 (fixation loss < 25%, false positive < 20%, and false negative < 33%) within 6 months before or after SS-OCT were included. Patients with a cataract of grade 3 or higher (Emery-Little classification), other types of glaucoma such as primary angle closure glaucoma, pseudoexfoliation glaucoma, steroid-induced glaucoma, uveitic glaucoma, or congenital glaucoma were excluded. Patients with diseases that may affect the sensitivity of the visual field or clarity of en-face images, such as retinal diseases and optic nerve diseases, or with epiretinal membrane (ERM), were also excluded. There was no exclusion criterion to determine whether an evaluation of NFBs similar to non-myopic eyes could be performed on myopic eyes, which often have tigroid fundus, using spherical equivalents.

Method

SS-OCT and acquisition of en-face images

The examinations of SS-OCT were performed after the pupil was dilated with 0.5% tropicamide and phenylephrine (Mydrin-P; Santen, Osaka, Japan). SS-OCT has an 8-μm depth of resolution in tissue and a 20-μm transverse resolution, and can acquire 100,000 axial scans per second. Imaging was performed with 512×256 axial scans and 6×6-mm cube scans centered at the fovea and optic disc. After flatten processing against the inner limiting membrane (ILM), en-face images were obtained by 6×6-mm 3D scan using the EnView imaging software. At this time, depth from ILM was determined at the point where the nerve fiber bundles (NFBs) were most clearly distinguishable in each case. In order to delineate the defective region and the remaining NFB region in the acquired image, brightness, contrast, and γ value were corrected appropriately. The two images (macula and optic disc) were superimposed using Photoshop (Adobe Systems, San Jose, CA, USA) based on the positions of large blood vessels. Using this method, we were able to observe the retinal nerve fibers in the outermost layer at the macula and periphery of the optic disc.

Creation of estimated visual field

After identifying the corresponding HFA10-2 locations on the acquired en-face images

with RGC displacement [10,11], the existence of NFBs at each point was evaluated by three examiners who were blinded to the visual field data. When judgments differed among examiners, the issue was decided by majority vote. The estimated visual field differs by two gradations from a field in which NFB defects are present, i.e., we only estimated whether there was a decline in sensitivity, and did not estimate the degree of visual field sensitivity.

Comparison with HFA10-2

Test points with <5%, <2%, and <1% of the probability plot in the total deviation (TD) and pattern deviation (PD) HFA10-2 were defined as points with visual field defects and compared between visual field defects and NFB defect

In addition, with reference to Koseki's HFA10-2 cluster [12], we investigated whether there was a difference in the degree of matching between clusters. Clusters are classified as shown in Fig 1; C1 corresponds to the papillomacular area identified in the preliminary study based on the correlation between the foveal threshold and the HFA10-2 examination point, C2 is the upper half of the visual field excluding the papillomacular area, and C3 is the lower half of the visual field excluding the papillomacular area.

Fig 1. Cluster classification. C1 is the papillomacular area, C2 is the upper half of the visual field excluding C1, and C3 is the lower half of the visual field excluding C1.

Statistical analysis

Statistical analysis was performed in Microsoft Excel 2016 using the BellCurve plugin (Social Survey Research Information Co. Japan). The κ coefficient was used to determine the degree of matching in judgement. Test points with defects in both the estimated visual field and actual visual field were defined as "positive," and test points with defects neither in the estimated visual field nor the actual visual field were defined as "negative." Test points with a defect only in the estimated visual field, but not in the actual visual field, were defined as "false positive." Test points with no defect in the estimated visual field, but with a defect in the actual visual field, were defined as "false negative." A total of 68 points were evaluated. Accuracy was determined based on the number of test points with positive and negative judgement. We classified subjects into two groups (spherical equivalent ≥ -6 diopters and < -6 diopters), and investigated whether there was a difference in the degree of matching. The intraclass correlation coefficient between examiners was determined. The Mann-Whitney U-test was used to compare κ coefficients. Statistical significance was defined as $p < 0.05$.

Results

The profiles of the 38 patients enrolled in this study are shown in Table 1. We recruited and included patients with early- to late-stage glaucoma in this study.

Table 1. Profiles of Patients Enrolled in this Study.

Patient (eyes/cases)	38/38
Age (years, range)	56.5 ± 9.7 (22 to 78)
Sex (female/male)	19/19
Spherical Equivalent (diopter)	–4.3 ± 4.0 (–11.375 to +2.625)
BCVA (LogMAR)	–0.049 ± 0.07 (–0.079 to 0.155)
BCVA	20/18 ± 20/140 (20/30 to 20/16)
HFA24-2 or 30-2 MD (dB)	–10.8 ± 6.2 (–0.3 to –23.5)
VFI (%)	67.5 ± 21.0 (20 to 98)
HFA10-2 MD (dB)	–12.1 ± 7.6 (–1.65 to –29.84)

Values represent means ± standard deviation.

POAG, primary open angle glaucoma; BCVA, best-corrected visual acuity; LogMAR, logarithm of the minimum angle of resolution; HFA, Humphrey Field Analyzer; VFI, visual field index; MD, mean deviation.

As shown in Fig 2A, an image was obtained by superimposing en-face images of the optic disc and macula areas. After overlaying the points corresponding to HFA10-2 locations

on the acquired en-face image with RGC displacement[10,11], the existence of NFBs was determined at each point (Fig 2B). The estimated visual field was created by turning the resultant image upside down (Fig 2C). The actual HFA10-2 grayscale, TD, and PD from a patient are shown in Fig 2D.

Fig 2. A. After superimposition of en-face images of the optic disc and macula area. B. After points corresponding to HFA10-2 locations were overlaid on the acquired en-face image with RGC displacement, the existence of NFBs was determined at each point. Filled circles indicate points where NFBs were absent, and open circles indicate points where NFBs were present. C. The estimated visual field (right) was created by turning the image at left upside down. D. Grayscale, TD, PD.

Figure 3 shows an example of an en-face image; Figure 3A shows a case in which the papillomacular area was damaged and only a few retinal nerve fiber layers (RNFLs) remained, and Fig 3B shows a case of diffuse RNFL defect.

Fig 3. A. A case of damage to the papillomacular area. B. A case of diffuse RNFL defect.

The average intraclass correlation coefficient between examiners was calculated for each case: the value was 0.94 ± 0.07.

When the test points of <5%, <2%, and <1% of the probability plot in TD and PD were defined as visual field defects, the κ coefficient at all 68 test points was 0.58, 0.64, and 0.66 in TD, respectively, and 0.68, 0.69, and 0.67 in PD (Table 2). There were no significant differences between TD and PD, in any of the probability plots. The accuracy (%) was, respectively, 82.6, 86.8, and 88.3 in TD and 87.7, 88.4, and 88.0 in PD. There were no significant differences between TD and PD, in any of the probability plots. In the group with spherical equivalent ≧ –6 diopters, the κ coefficients for <5%, <2%, and <1% were, respectively, 0.58, 0.62, and 0.63 in TD and 0.66, 0.67, and 0.65 in PD, whereas for the myopic group with spherical equivalent < –6 diopters, the values were 0.58, 0.69, and 0.71 in TD and 0.72, 0.71, and 0.71 in PD (Table 2). There was no difference between the two groups in the degree of matching. In the cluster analysis, the accuracy (%) in TD and PD for the <1% probability plot was 94.4 and 94.4 at C1, 84.5 and 83.3 at C2, and 89.5 and 90.3 at C3, respectively (Table 3). Accuracy was highest in the papillomacular area, and there was a tendency for the accuracy to be higher in the lower visual field than in the upper visual field.

Table 2. κ Coefficient and Accuracy (%), Total and by Spherical Equivalent.

κ Coefficient (Accuracy %)	TD < 1%	TD < 2%	TD < 5%	PD < 1%	PD < 2%	PD < 5%
Total	0.66 (88.3)	0.64 (86.8)	0.58 (82.6)	0.67 (88.0)	0.69 (88.4)	0.68 (87.7)
Spherical Equivalent ≥ –6 Diopters	0.63 (88.5)	0.62 (87.3)	0.58 (84.9)	0.65 (87.9)	0.67 (88.5)	0.66 (87.9)
Spherical Equivalent < –6 Diopters	0.71 (88.0)	0.69 (86.0)	0.58 (78.3)	0.71 (88.1)	0.71 (88.1)	0.72 (87.2)

Table 3. Accuracy (%), by cluster.

Accuracy (%)	TD < 1%	PD < 1%
Cluster 1	94.4	94.4
Cluster 2	84.5	83.3
Cluster 3	89.5	90.3

Discussion

In this report, we estimated the central 10-degree visual field based on the presence or absence of NFBs in en-face images of SS-OCT. This method achieved a high concordance with the results of actual HFA10-2 data. Various methods have been developed for

observing the RNFL and nerve fiber layer defect (NFLD): regular fundus photography, red-free fundus photography[13], scanning laser ophthalmoscopy (SLO) [14], adaptive optics SLO (AO-SLO) [15], scanning laser polarimetry (GDx) [16], Heidelberg Retinal Tomography (HRT) [17], and OCT. Fundus photography is advantageous because image acquisition is easy. However, observation of NFBs is restricted in patients with tigroid fundus. As with fundus photography, red-free fundus photography can be easily acquired, and the detection rate of NFLD is higher than that of ordinary fundus photography [18]; however, it is not well suited to quantitative analysis. Jung et al. [19] compared red-free fundus photography with en-face images of OCT and reported that NFLD detected with red-free light was also detectable in en-face images of OCT; therefore, the two methods are considered to have the same diagnostic utility. Although AO-SLO can obtain high-resolution images, it is not well suited to quantitative analysis. Parametric analysis with HRT, GDx, and OCT are quantitative and objective tests to evaluate the presence of glaucomatous changes because they compare patient data against a database of normal volunteers; these methods are advantageous in terms of objectivity, quantitativeness, and reproducibility. However, these parametric analyses may yield inaccurate results in patients with high myopia [20].

On the other hand, the en-face image of OCT is qualitative but directly observes the

retinal nerve fibers. The en-face image of OCT is not a comparative test, so there are no restrictions on the status of the subjects. As we showed in this study, the accuracy of the estimated visual field did not differ in highly myopic patients; therefore, we consider evaluation of the RNFL and estimation of the visual fields using en-face images to be useful even in cases of high myopia, which as noted above limits the application of parametric analysis using OCT. Hood et al. [21] described four advantages of en-face images over a RNFL thickness map in OCT, as follows: First, there are few segmentation errors, because segmentation only depends on identifying the border between the vitreous and the ILM. Second, blood vessels are easily distinguished from local intact bundles of the RNFL. Third, abnormal regions of RNFL can vary in reflectance intensity and thickness. Unlike measures of RNFL thickness, OCT en-face images of reflectance intensity are affected by both kinds of abnormalities. Fourth, the RNFL thickness map segmentation algorithms for identification of layer boundaries include local spatial averaging, which acts as a low-pass filter and results in loss of spatial detail. Miura et al. [22] reported that the NFLD angle, a new parameter based on the en-face image, has an intra-class correlation coefficient of 0.988, and correlates with the severity of glaucoma (MD and upper and lower TD), making the en-face image useful for glaucoma diagnosis.

We believe that the prominent advantage of the evaluation of the glaucomatous optic disc and retinal changes using en-face images is that RNFL, including the macular area, can be more clearly visualized. In general examinations such as the visual field test and OCT, emphasis has been placed on how to detect abnormalities; by contrast, we believe that our method is sensitive to detecting remaining RNFL in cases of various stages of glaucoma, especially advanced glaucoma. Moreover, the greatest advantage of the en-face image of OCT is the ability to visualize the presence or absence of residual RNFL in the papillomacular area, and further, to estimate visual field from the status of RNFL. As shown in this study, accuracy was highest in the papillomacular cluster (Table 3), so this is an excellent method for evaluating the visual field corresponding to this fundus area. Asaoka et al. [23] reported that visual acuity correlated with the sensitivity of HFA10-2, and that the correlation coefficient was high in the papillomacular area, which tended to remain until the end stage. Therefore, the papillomacular area is useful for estimation of VR-QOL. In this study, we used RGC displacement [10,11] to overlay the points corresponding to HFA 10-2 test locations onto the acquired en-face image. RGC is displaced in the macular area [24]. Ohkubo et al. [10], who used RGC displacement, reported a correlation between GCC thickness and all 68 test points corresponding to HFA 10-2. They also reported a high correlation, especially at the four central points,

and argued that this method is necessary for assessing the structure–function relationship in the macula. We believe that the two central points included in the papillomacular area exhibited a high degree of matching because RGC displacement was used.

Regarding estimation of the visual field, Takahashi et al. [25] estimated the central 10-degree visual field from macular OCT using the correlation between RNFL thickness and visual field sensitivity in the 68 test points; they reported a high correlation with the observed HFA10-2 visual field, and that their method would be useful for patients in which visual field examinations are difficult. An advantage of our method compared to their method is detection of residual NFB in advanced cases, which may be a floor effect in OCT resul[illegible]They stated that the nasal area, which is susceptible to glaucomatous damage, had a low correlation between RNFL thickness and visual field sensitivity due to the floor effect. In addition, our method may be more useful for highly myopic patients because the previous study excluded subjects with myopia < –8 diopters.

Some studies have reported correlations between retinal inner layer thickness and visual field sensitivity in the macula [10,26]; to date, however, there have been no reports of a relationship between the en-face image of OCT and visual field sensitivi[illegible]Because the degree of matching with the visual field was higher in the TD <1% probability plot

than the <5% probability plot, the point of NFB defect in the en-face image suggests a large decrease in the sensitivity of visual function[illegible]

Future studies should seek to clarify the relationship between retinal sensitivity and areas with NFB defects in the en-face imag[illegible]s well as the relationship between the foveal threshold and visual acuity in patients whose papillomacular nerve fibers remain. In addition, it is necessary to devise a more accurate estimation method. Specifically, to minimize the influence of artifacts on clinicians' judgment, it might be possible to set a reference by binarizing the image, in order to add information about the thickness of the retinal inner layer of the macula to the en-face imag[illegible]t may also be necessary to consider whether the degree of matching can be improved by correcting the size of the index projected on the fundus due to the axial length, as well as overlaying the index using RGC displacement considering the disc inclination.

This study has several limitations. First, the judgment of NFB defects is subjective. However, high intraclass correlation coefficients were observed in this study, and examiner variation was small. Second, regardless of axial length, the same figure of RGC displacement is overlaid on the acquired en-face image. The size of an object projected onto the fundus differs depends on the axial length; however, because this was a retrospective study, data on axial length was insufficient, and this was not corrected.

Third, we did not consider the inclination of the disc. Fourth, en-face images cannot be obtained in cases with ERM, so our method cannot be applied to all cases. Fifth, among the patients in this study, there was some bias: specifically, only a few cases had disorders of the papillomacular area, whereas many more cases had defects in the upper visual field defect than in the lower visual field. Therefore, it would be desirable to perform additional studies with a larger number of cases and investigate each stage of glaucoma.

In summary, using the OCT en-face image, we could estimate the central 10-degree visual field, and we obtained a high degree of concordance with actual HFA10-2 data. This method may be useful for detecting functional abnormalities based on structural abnormalities.

Acknowledgments

Author Contributions

Conceptualization: Ryu Iikawa, Takeo Fukuchi.

Data curation: Ryu Iikawa, Daiki Miyamoto, Takeo Fukuchi.

Formal analysis: Ryu Iikawa, Kiyoshi Yaoeda, Masaaki Seki, Takeo Fukuchi.

Investigation: Ryu Iikawa, Daiki Miyamoto.

Methodology: Ryu Iikawa, Takeo Fukuchi.

Project administration: Takeo Fukuchi.

Resources: Ryu Iikawa, Tetsuya Togano, Yuta Sakaue, Aki Suetake, Ryoko Igarashi, Daiki Miyamoto, Kiyoshi Yaoeda, Masaaki Seki, Takeo Fukuchi.

Software: Ryu Iikawa, Daiki Miyamoto.

Supervision: Takeo Fukuchi.

Validation: Ryu Iikawa, Tetsuya Togano, Yuta Sakaue, Aki Suetake, Ryoko Igarashi.

Visualization: Ryu Iikawa.

Writing – original draft: Ryu Iikawa.

Writing – review & editing: Kiyoshi Yaoeda, Masaaki Seki Takeo Fukuchi.

Reference

1. Quigley HA, Dunkelberger GR, Green WR. Retinal ganglion cell atrophy correlated with automated perimetry in human eyes with glaucoma. Am J Ophthalmol. 1989;107(5): 453–464.

2. Japan Glaucoma Society. The Japan Glaucoma Society Guidelines for Glaucoma (4th Edition). Nippon Ganka Gakkai Zasshi. 2018;122(1): 5–53. Japanese.

3. Rao HL, Begum VU, Khadka D, Mandal AK, Senthil S, Garudadri CS. Comparing glaucoma progression on 24-2 and 10-2 visual field examinations. PLoS One. 2015;10(5): e0127233.

4. Sun Y, Lin C, Waisbourd M, Ekici F, Erdem E, Wizov SS, et al. The Impact of Visual Field Clusters on Performance-based Measures and Vision-Related Quality of Life in Patients With Glaucoma. Am J Ophthalmol. 2016;163: 45–52.

5. Wang DL, Raza AS, de Moraes CG, Chen M, Alhadeff P, Jarukatsetphorn R, et al. Central Glaucomatous Damage of the Macula Can Be Overlooked by Conventional OCT Retinal Nerve Fiber Layer Thickness Analyses. Transl Vis Sci Technol. 2015;4(6): 4.

6. Traynis I, De Moraes CG, Raza AS, Liebmann JM, Ritch R, Hood DC. Prevalence and nature of early glaucomatous defects in the central 10° of the visual field. JAMA Ophthalmol. 2014;132(3): 291–297.

7. Sullivan-Mee M, Karin Tran MT, Pensyl D, Tsan G, Katiyar S. Prevalence, Features, and Severity of Glaucomatous Visual Field Loss Measured With the 10-2 Achromatic Threshold Visual Field Test. Am J Ophthalmol. 2016;168: 40–51.

8. Park SC, Kung Y, Su D, Simonson JL, Furlanetto RL, Liebmann JM, et al. Parafoveal scotoma progression in glaucoma: humphrey 10-2 versus 24-2 visual field analysis. Ophthalmology. 2013;120(8): 1546–1550.

9. Grillo LM, Wang DL, Ramachandran R, Ehrlich AC, De Moraes CG, Ritch R, et al. The 24-2 Visual Field Test Misses Central Macular Damage Confirmed by the 10-2 Visual Field Test and Optical Coherence Tomography. Transl Vis Sci Technol. 2016;5(2): 15.

10. Ohkubo S, Higashide T, Udagawa S, Sugiyama K, Hangai M, Yoshimura N, et al. Focal relationship between structure and function within the central 10 degrees in glaucoma. Invest Ophthalmol Vis Sci. 2014;55(8): 5269–5277.

11. Raza AS, Cho J, de Moraes CG, Wang M, Zhang X, Kardon RH, et al. Retinal ganglion cell layer thickness and local visual field sensitivity in glaucoma. Arch Ophthalmol. 2011;129(12): 1529–1536.

12. Koseki N, Araie M, Yamagami J, Suzuki Y. Sectorization of central 10-deg visual field in open-angle glaucoma. An approach for its brief evaluation. Graefes Arch Clin Exp

Ophthalmol. 1995;233(10): 621–626.

13. Hoyt WF, Frisén L, Newman NM. Fundoscopy of nerve fiber layer defects in glaucoma. Invest Ophthalmol. 1973;12(11): 814–829.

14. Uchida H, Tomita G, Onda E, Sugiyama K, Kitazawa Y. Relationship of nerve fiber layer defects and parafoveal visual field defects in glaucomatous eyes. Jpn J Ophthalmol. 1996;40(4): 548–553.

15. Takayama K, Ooto S, Hangai M, Arakawa N, Oshima S, Shibata N, et al. High-resolution imaging of the retinal nerve fiber layer in normal eyes using adaptive optics scanning laser ophthalmoscopy. PLoS One. 2012;7(3): e33158.

16. Badalà F, Nouri-Mahdavi K, Raoof DA, Leeprechanon N, Law SK, Caprioli J. Optic disk and nerve fiber layer imaging to detect glaucoma. Am J Ophthalmol. 2007;144(5): 724–732.

17. Malinovsky VE. An overview of the Heidelberg Retina Tomograph. J Am Optom Assoc. 1996;67(8): 457–467.

18. Bae HW, Lee N, Kim CY, Choi M, Hong S, Seong GJ. Comparison of three types of images for the detection of retinal nerve fiber layer defects. Optom Vis Sci. 2015;92(4): 500–505.

19. Jung JH, Park JH, Yoo C, Kim YY. Localized Retinal Nerve Fiber Layer Defects in

Red-free Photographs Versus En Face Structural Optical Coherence Tomography Images. J Glaucoma. 2018;27(3): 269–274.

20. Fang Y, Zhang HQ, Qiao RH, Yao XY, Pan YZ, Li M. Effectiveness of Glaucoma Diagnostic Parameters from Spectral Domain-Optical Coherence Tomography of Myopic Patients. Chin Med J (Engl). 2018;131(15): 1819–1826.

21. Hood DC, Fortune B, Mavrommatis MA, Reynaud J, Ramachandran R, Ritch R, et al. Details of Glaucomatous Damage Are Better Seen on OCT En Face Images Than on OCT Retinal Nerve Fiber Layer Thickness Maps. Invest Ophthalmol Vis Sci. 2015;56(11): 6208–6216.

22. Miura N, Omodaka K, Kimura K, Matsumoto A, Kikawa T, Takahashi S, et al. Evaluation of retinal nerve fiber layer defect using wide-field en-face swept-source OCT images by applying the inner limiting membrane flattening. PLoS One. 2017;12(10): e0185573.

23. Asaoka R. The relationship between visual acuity and central visual field sensitivity in advanced glaucoma. Br J Ophthalmol. 2013;97(10): 1355–1356.

24. Sjöstrand J, Popovic Z, Conradi N, Marshall J. Morphometric study of the displacement of retinal ganglion cells subserving cones within the human fovea. Graefes Arch Clin Exp Ophthalmol. 1999;237(12): 1014–1023.

25. Takahashi M, Omodaka K, Maruyama K, Yamaguchi T, Himori N, Shiga Y, et al. Simulated visual fields produced from macular RNFLT data in patients with glaucoma. Curr Eye Res. 2013;38(11): 1133–1141.

26. Honjo M, Omodaka K, Ishizaki T, Ohkubo S, Araie M, Nakazawa T. Retinal Thickness and the Structure/Function Relationship in the Eyes of Older Adults with Glaucoma. PLoS One. 2015;10(10): e0141293.

 Click here to download Figure Fig1.tif

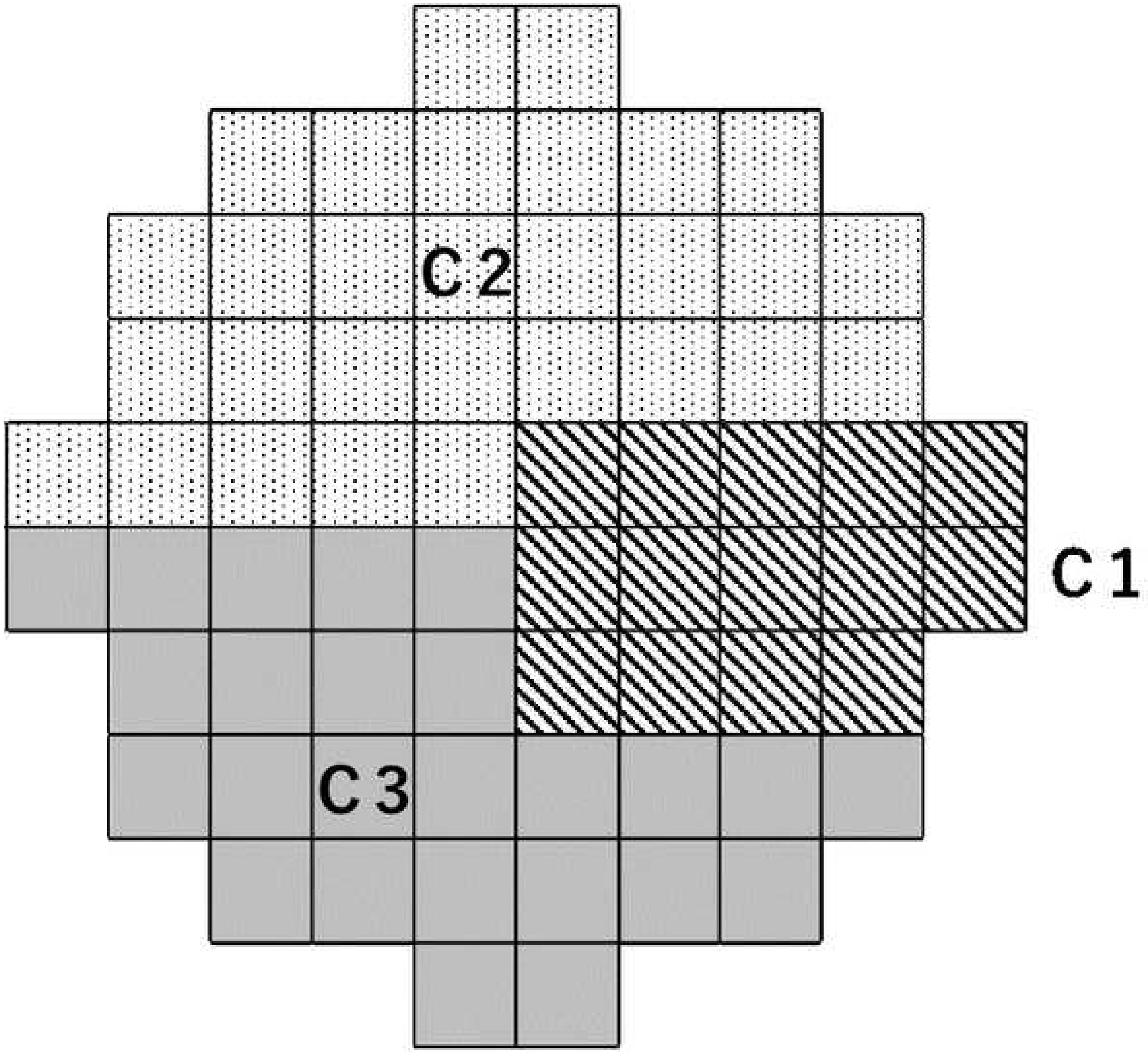

 Click here to download Figure Fig2A.tif

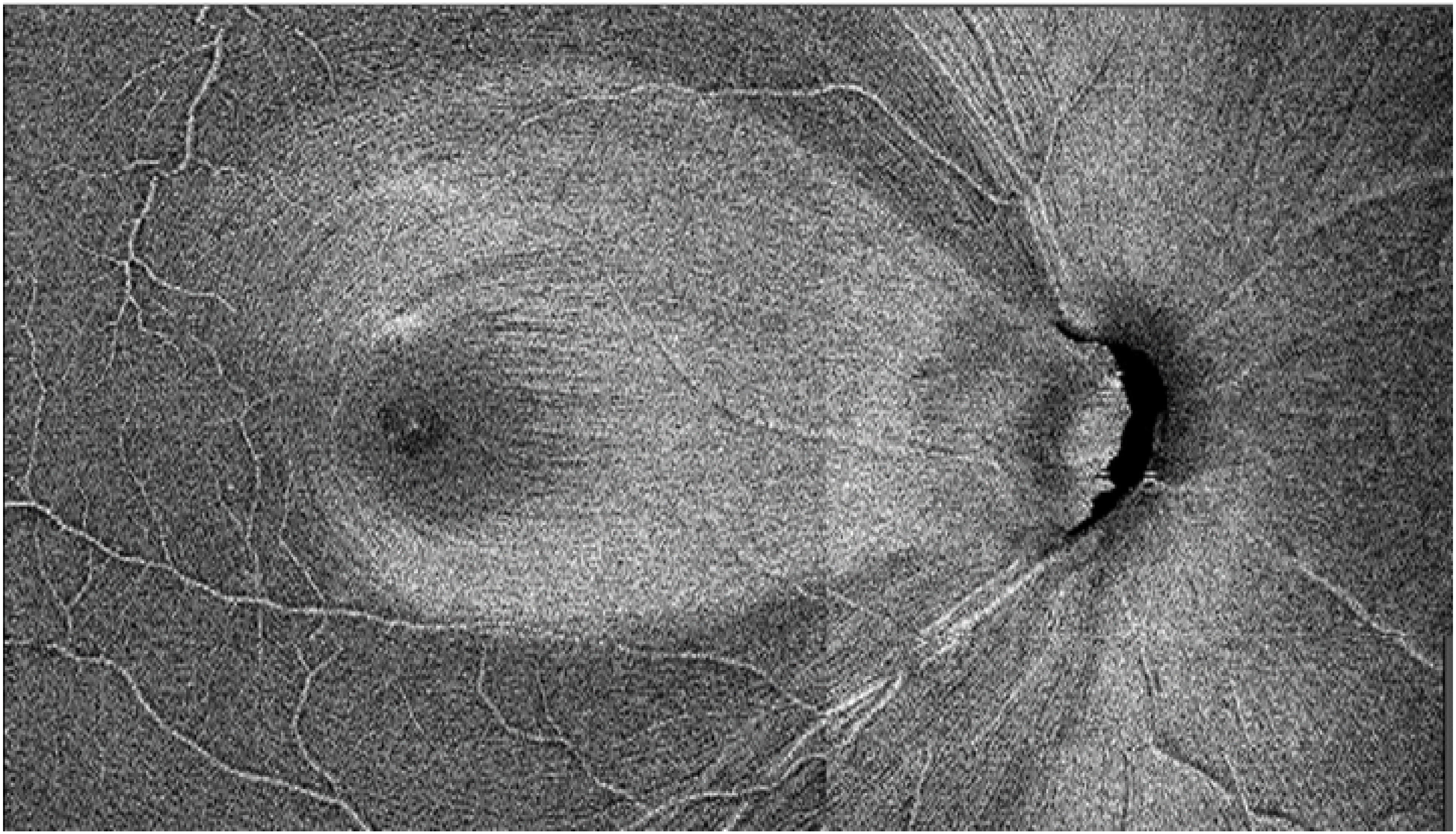

Figure

Click here to download Figure Fig2B.tif

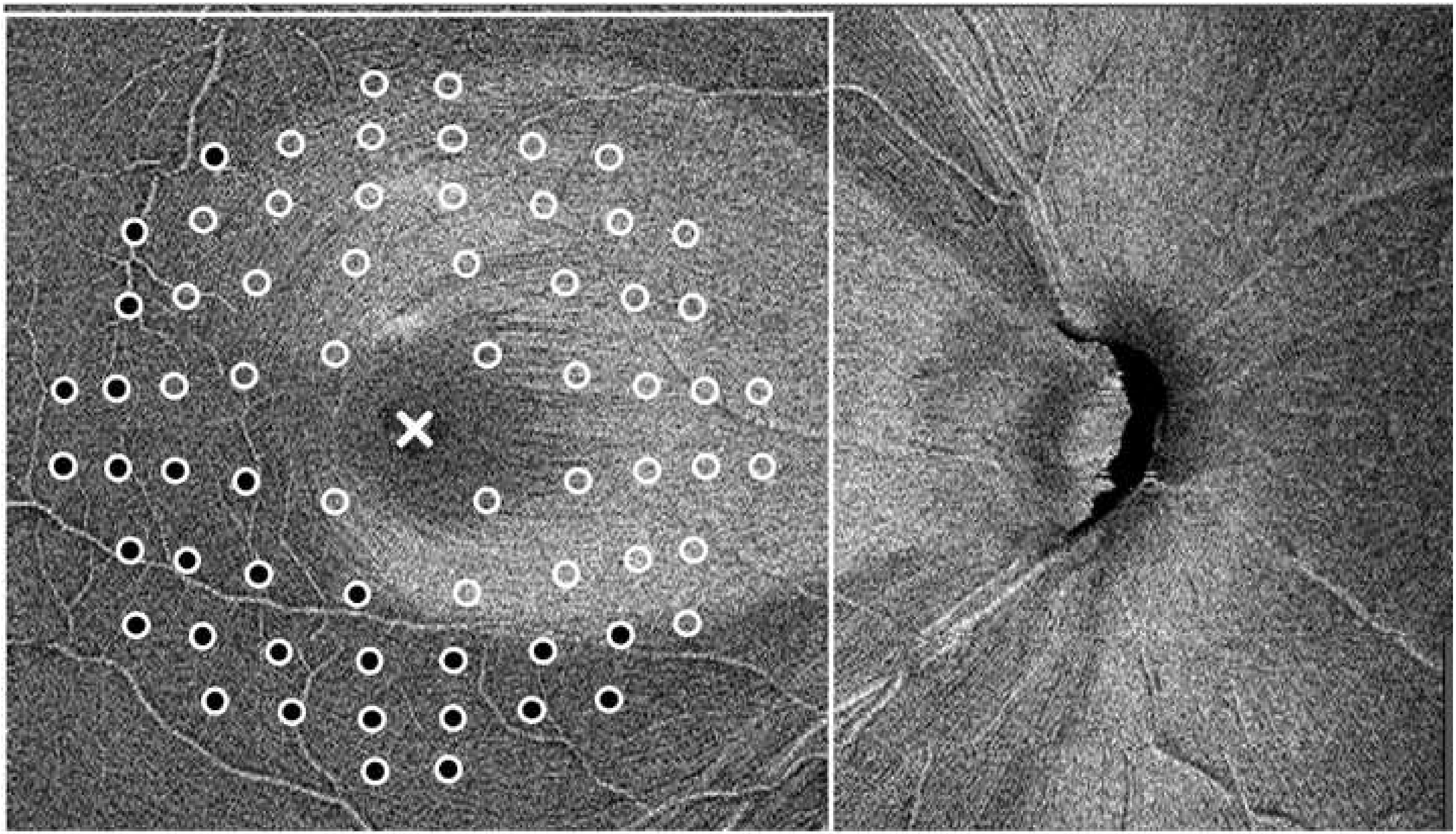

 Click here to download Figure Fig2C.tif

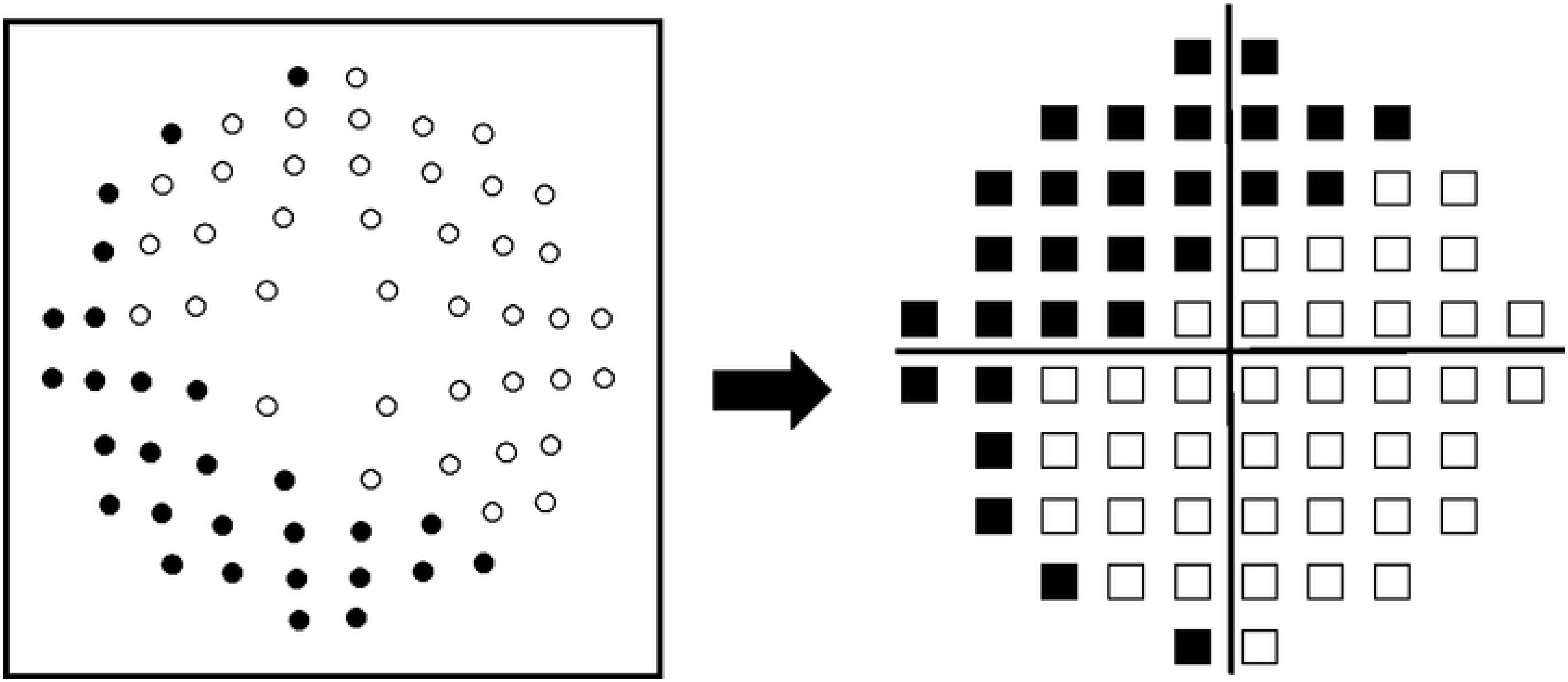

Figure

Click here to download Figure Fig2D.tif

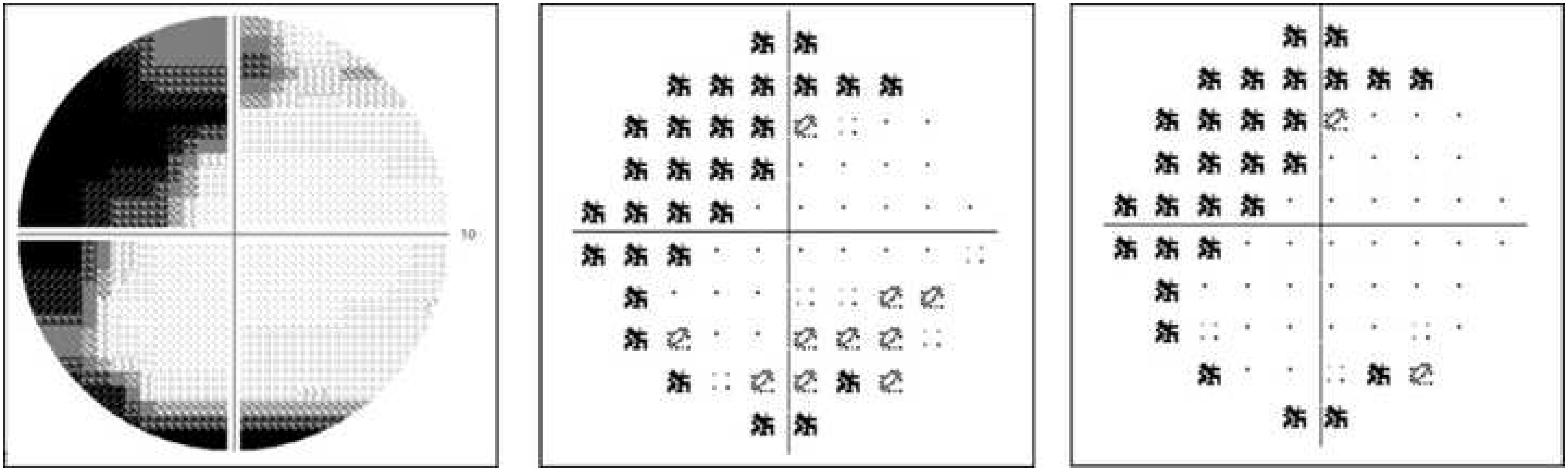

Click here to download Figure Fig3A.tif

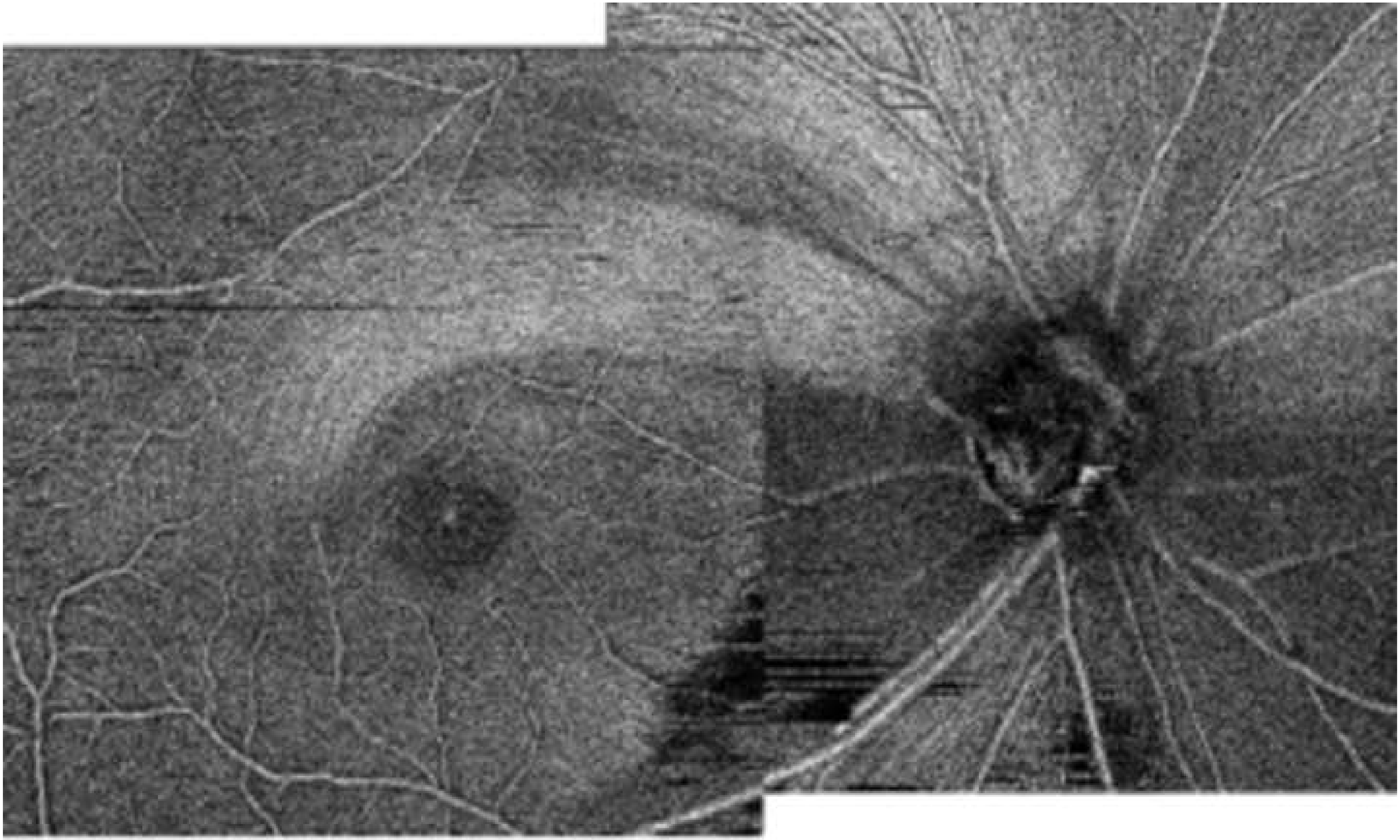

 Click here to download Figure Fig3B.tif

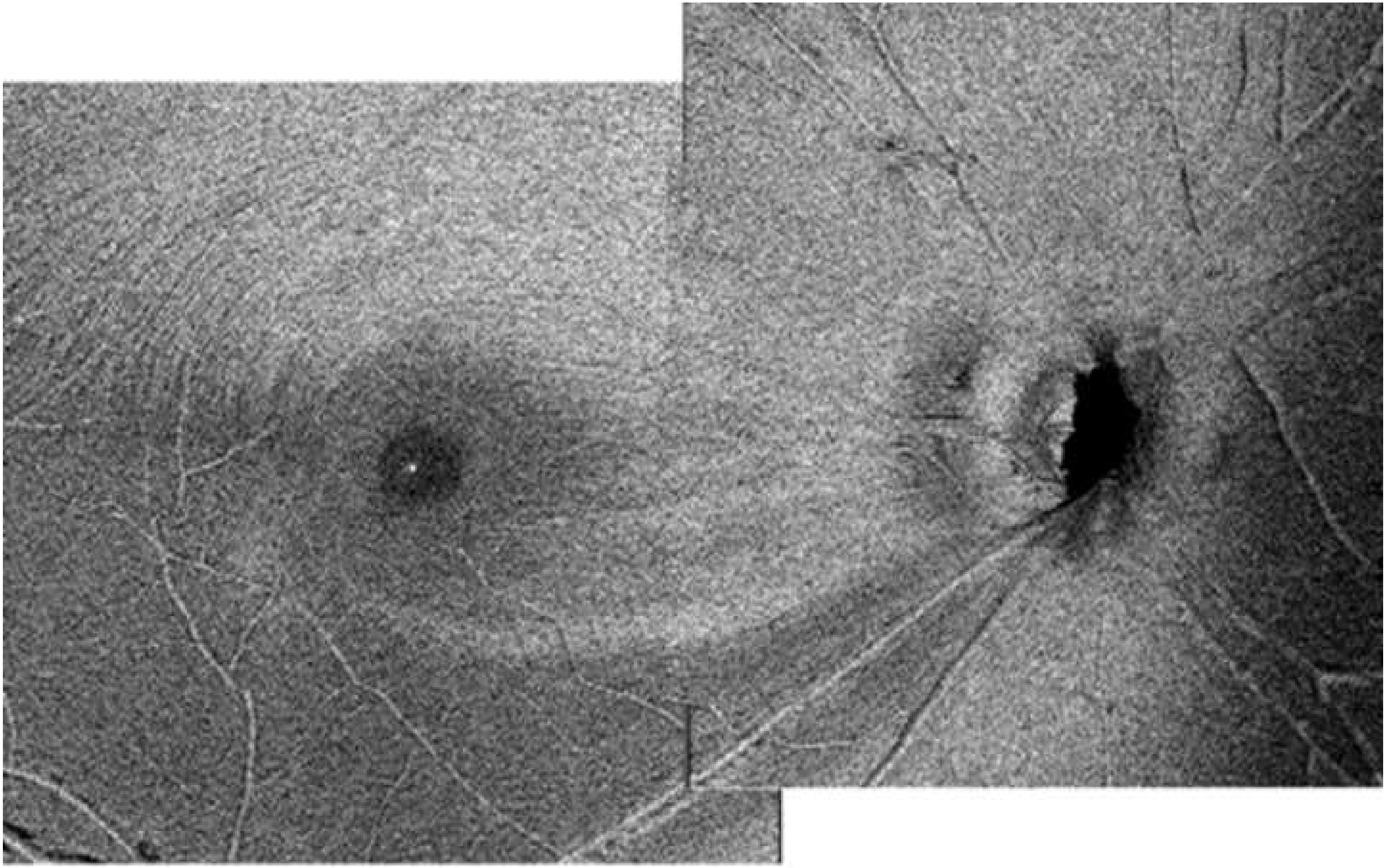

Supplement: Attachment — Submitted filename: PONE-D-19-21988_reviewer .pdf [file pone.0229867.s001.pdf]
