# Supplementary material for: Estimation of the central 10-degree visual field using en-face images obtained by optical coherence tomography
Source: PLoS One. 2020 Mar 5;15(3):e0229867. doi: 10.1371/journal.pone.0229867 (PMC7058317; doi:10.1371/journal.pone.0229867)
Supplement: Attachment — Submitted filename: Response to Reviwers.docx [file pone.0229867.s002.docx]

Dr. Sanjoy Bhattacharya

Academic Editor

*PLOS ONE*

November 26, 2019

Dear Dr. Bhattacharya,

Subject: Estimation of the central 10-degree visual field using en-face images obtained by optical coherence tomography” **[PONE-D-19-21988] - [EMID:** **b9a3d83ef5bec8b9]**.

Thank you for your email dated October 24, 2019, enclosing the reviewers’ comments. We have carefully reviewed the comments and have revised the manuscript accordingly. Our responses are given in a point-by-point manner below. Changes to the manuscript are indicated with highlighted text.

We look forward to hearing from you regarding our submission. We would be glad to respond to any further questions and comments that you may have.

Sincerely,

Ryu Iikawa, MD

Division of Ophthalmology and Visual Science, Graduate School of Medical and Dental Sciences,

Niigata University

Reviewer’s Comments to Author (in italics, followed by our responses in regular typeface)

Response to Journal Requirements:

*> 1. When submitting your revision, we need you to address these additional requirements.*

*Please ensure that your manuscript meets PLOS ONE's style requirements, including those for file naming. The PLOS ONE style templates can be found at*

[*http://www.journals.plos.org/plosone/s/file?id=wjVg/PLOSOne_formatting_sample_main_body.pdf*](http://www.journals.plos.org/plosone/s/file?id=wjVg/PLOSOne_formatting_sample_main_body.pdf) *and http://www.journals.plos.org/plosone/s/file?id=ba62/PLOSOne_formatting_sample_title_authors_affiliations.pdf*

Thank you for pointing out our formatting errors. We added the table title and footnotes as indicated below. These changes to the manuscript are highlighted in the text (line 184, 219 and 212).

*> 2. Please provide additional details regarding participant consent. In the Methods section, please ensure that you have specified (1) whether consent was informed and (2) what type you obtained (for instance, written or verbal). If your study included minors, state whether you obtained consent from parents or guardians. If the need for consent was waived by the ethics committee, please include this information.*

Thank you very much for the comment. This is a retrospective study. Information about the research was made public on the Niigata University website, and consent was obtained by giving subjects the opportunity to opt out. These changes to the manuscript are highlighted in the text (line82-84).

*>3. We noticed you have some minor occurrence of overlapping text with the following previous publication(s), which needs to be addressed:*

*https://iovs.arvojournals.org/article.aspx?articleid=2451212&resultClick=1*

Thank you for your comment. We changed the sentences in discussion line 244-249. These changes to the manuscript are highlighted in the text.

**Response to Reviewer #1:**

Thank you for taking the time to review our paper. We have responded to each of your points below.

Major comments:

*>1. This research is strong, potentially clinically applicable, and original. There are many studies that investigate prediction of glaucomatous visual field progression, but limited research has been done in estimating defects based on nerve fiber layer analysis.*

*>2. The manuscript is technically thorough, and the included data supports the initial research question and conclusions. Statistical analysis was thorough and accurate.*

Thank you for your comment. Some previous reports have shown a correlation between nerve fiber bundle (NFB) defects in en-face images and visual field defects in HFA10-2, but this is the first study to examine the degree of matching for each test point by Kappa coefficients.

*>3. Form of consent should be included due to human participants.*

Thank you for your comment. The same was shown in “Journal Requirements”. These changes to the manuscript are highlighted in the text (line82-84).

*>4. Discussion: Cases were biased toward more visual field defects in the upper visual field and few in the papillomacular bundle, yet the visual field cluster with lowest estimation accuracy was the upper cluster and the highest accuracy was the papillomacular bundle. The authors should address this more in the discussion, as the upper visual field defect was the most common and least accurate, and the papillomacular bundle defect was the most accurate and the least common. The authors should also address a possible explanation as to why there is a discrepancy between upper and lower visual field defect estimation accuracy.*

Thank you very much for your insight about the point. According to your suggestion, we added the sentences in discussion (line272-283 with highlight).

Minor Comments:

*>1. Abbreviation RGC should be defined on line #23.*

Thank you for your comment. We defined RGC: retinal ganglion cell (line23 with highlight).

*>2. Abbreviation NFB should be defined on line #69. It was addressed in the abstract, but this is the first use of the abbreviation in the manuscript.*

Thank you for your comment. We defined NFB: nerve fiber bundle (line74-75 with highlight).

*>3. “Goldman” on line #82 should be corrected to Goldmann.*

Thank you for your comment. We collected the word (line90 with highlight).

*>4. The word “defect” on line #305 should be deleted.*

Thank you for your comment. We deleted the word (line359).

**Response to Reviewer #2:**

Thank you for taking the time to review our paper. We have answered each of your points below.

*>The rational of this study is of a great interest because of the ability to provide more accurate diagnosis of glaucomatous defect, therefore better monitoring of the disease progression within the macula.*

*However, the estimation was based on the qualitative analysis (en face images of the RNFL bundles), meaning that the prediction would be about locations and extent of the perimetric defect. This concept should be emphasized across the manuscript because one may think that this estimation is about the depth of perimetric defect. Looking at the residual of the RNFL does not necessarily mean there would a certain depth of defect.*

Thank you for your comment. As you pointed out, this method estimates the location and extent of the visual field defects and cannot estimate the depth of defects. Although it was described in the method section (line 130-131), we also described in the discussion (line 330-331 with highlight) and limitation (line 348-349 with highlight).

*>1. A careful review of the literature is needed. Here are some examples of studies that used en face images with perimetry within the macula:*

*Hood DC, Fortune B, Mavrommatis MA, et al. Details of Glaucomatous Damage Are Better Seen on Oct En Face Images Than on Oct Retinal Nerve Fiber Layer Thickness Maps. Invest Ophthalmol Vis Sci 2015;56:6208-16.*

*Alluwimi MS, Swanson WH, Malinovsky VE, King BJ. Customizing Perimetric Locations Based on En Face Images of Retinal Nerve Fiber Bundles with Glaucomatous Damage. Translational Vision Science & Technology 2018;7:5-5.*

*Alluwimi MS, Swanson WH, Malinovsky VE, King BJ. A Basis for Customising Perimetric Locations within the Macula in Glaucoma. Ophthalmic Physiol Opt 2018;38:164-73.*

*Other studies regarding Quality of lif in glaucoma:*

*Goldberg I, Clement CI, Chiang TH, et al. Assessing Quality of Life in Patients with Glaucoma Using the Glaucoma Quality of Life-15 (Gql-15) Questionnaire. J Glaucoma 2009;18:6-12.*

*Garg A, Hood DC, Pensec N, et al. Macular Damage, as Determined by Structure-Function Staging, Is Associated with Worse Vision-Related Quality of Life in Early Glaucoma. Am J Ophthalmol 2018;194:88-94.*

We really appreciate the suggestion and have thoroughly read the suggested papers. According to Reviewer’s suggestion, we added the second and third literature to the references. (The first literature was already included in the references.)

*>2. Line 49: Please define what HFA 24-2. This will lead the readers to understand the following text.*

Thank you for your comment. We defined HFA 24-2: Humphrey Field Analyzer, and added the following sentence: HFA 24-2 program in which test points are separated by 6 degrees within the central 24 degrees (line 48-50 with highlight).

*>3. Line 52: References are needed. above are two examples.*

Thank you for your suggestion. We added the two literatures to the references (line 53 with highlight).

*>4. Line 57: the word "fundus" could be deleted.*

Thank you for your comment. We deleted the word (line 63).

*>5. Line 67: It should be mentioned that ±10° of the visual field.*

Thank you for your comment. We mentioned that test points of HFA10-2 are separated by 2-degree within the central 10-degree (line55-56 with highlight). This sentence means the same test points as HFA10-2 (line 72-73 with highlight).

*>6. Line 68: What is the justification for the time (few seconds)? Are there references? or do you have data support the time consumption that was mentioned?*

Thank you for your comment. The OCT we used in this study (DRI OCT-1 Atlantis; Topcon, Tokyo, Japan) can acquire 100,000 axial scans per second. And OCT imaging was performed with 512×256 axial scans (i.e. 131,072 scans), thus data acquisition alone only requires 1.31072 sec. in theory (line 111-113).

*>7. Line 70: Could you please carefully review the literature (examples are above) and rewrite the sentence to clarify what is different in this study?*

Thank you for your comment. We changed the sentences to clarify what is different in this study (line 74-76 with highlight).

*>8. Line 94: the sentence needs to be rephrased, it is not clear.*

Thank you for your comment. We rephrased the sentence: “In order to determine whether similar results could be obtained for highly myopic and eyes that were not highly myopic, we set no exclusion criteria for the spherical equivalent.” (line 103-105 with highlight).

*>9. In the method section, it was not clear how many visits were used to assess the 10-2 test.*

Thank you for your comment. Only single HFA 10-2 data (i.e. a single visit) was used for analysis. We added a sentence in the method section to make it clear (line 93-94 with highlight).

*>10. Line 127: could you clarify more the criterion for defining test points that are less 5%, 2% and 1% of abnormality, for example, 2 consecutive points? with the last consecutive visits?*

Thank you for your comment. Test points with <5%, <2%, and <1% of the “probability plot” are automatically outputted as results of HFA 10-2 on Humphrey Field Analyzer, but not what we originally defined in this study. They are not related to whether the points are consecutive or not. As we mentioned earlier, only single HFA 10-2 data was used for a patient in this study. We added the sentence about this as the 7th limitation (line 359-362 with highlight).

*>11. In the statistical analysis, it was very nice explaining how estimated versus actual defective points was categorized. However, it was not clear what the statistical test was used, which was the main question in the study. The statistical analysis used was for k coefficient.*

Thank you for your comment. We added sentences in statistical analysis section (line 159-165 with highlight). The κ coefficient was calculated using the positive, negative, false positive, and false negative numbers we defined according to the following formula: [2×{(positive×negative)−(false positive×false negative)}/{(positive+false positive)×(ngative+false positive)+(negative+false negative)×(positive+false negative)}] The accuracy and κ coefficient were determined for each patient and the average of 38 patients was calculated.

The Mann-Whitney U-test was used to compare κ coefficients and accuracy.

*>12. Line 169: Why would gray scale needed? Because it was mentioned that the estimation was about whether there was a defect or not. Gray scale is more about the degree of defect which was not estimated.*

Thank you for your comment. We now agree with your comment, thus omitted it.

*>13. Line 211 to 227: this is about literature review about different imaging modules, which could be briefly mentioned in the introduction. In discussion, this information disrupted the flow of the text.*

Thank you for your comment. According to the helpful suggestion, we moved the part to introduction and changed the sentences accordingly (line 58-62 with highlight).

*>14. It was frequently stated that different imaging modules do not provide quantitative analysis; en face view does not either.*

We totally agree with your comment. Thus, we added several sentences about the challenges to quantitation (line 292-293, 331-337 with highlight) so that readers will not misunderstand that en face view imaging is a quantitative analysis.

*>15. Line 229: rephrase the sentence.*

Thank you for your comment. We rephrase the sentence: “Because the en-face image does not rely on comparisons to a normal database, it can be adapted to anyone.” (line 233-234 with highlight)

*>16. Line 231: Why would high myopia affect central visual field? Are there references?*

Thank you for your comment. We don’t suggest that high myopia affect the central visual field. We added the sentences to prevent readers from misunderstanding. “We grouped the patients into two groups to evaluate whether our methods for estimating the field of view is useful even for patients in whom glaucoma is difficult to diagnose because NFLD may be difficult to evaluate by normal fundus photography due to tigroid fundus, or the use of OCT may be restricted due to severe myopia. (line 234-238 with highlight).

*>17. Line 266: Are there references supporting your references? did compare displaced versus non-displaced ganglion cell bodies?*

Thank you for your comment. “RGC displacement” is a method to correlate RGC location and test points of HFA 10-2, which was developed by Ohkubo et al. (Ref #10). RGC displacement here in this manuscript is not a histological term (e.g. displaced RGC or displaced amacrine cells).

*>18. Line 275: could you please show an example to clarify the idea more for the audience?*

Thank you for your comment. We added a figure in Fig 4: a case residual NFB is detected more clearly with the en-face image (line 317-319 with highlight).

*>19. Line 282 to 284 is not clear. Does talk about the degree of sensitivity?*

Thank you for your comment. En-face image can’t estimate degree of sensitivity, but we talk about the relationship between the en-face image and visual field sensitivity in these sentences. To make the point clear, we added the discussion about sensitivity referring to suggested papers (line 322-331 with highlight).

*>20. Line 285 and 286: There are studies, as mentioned earlier.*

Thank you for your comment. According to the suggestion, we changed the sentences (line 322-331 with highlight).

*>21. Line 291: There were actually published work, here are some examples:*

*Gardiner SK, Demirel S, Reynaud J, Fortune B. Changes in Retinal Nerve Fiber Layer Reflectance Intensity as a Predictor of Functional Progression in Glaucoma. Invest Ophthalmol Vis Sci 2016;57:1221-7.*

*Ashimatey BS, King BJ, Burns SA, Swanson WH. Evaluating Glaucomatous Abnormality in Peripapillary Optical Coherence Tomography Enface Visualisation of the Retinal Nerve Fibre Layer Reflectance. Ophthalmic Physiol Opt 2018;38:376-88.*

Thank you for your comment. According to the helpful comment, we changed the sentence on line331-337 with highlight and cited those two studies.
